# Species and sex-specific chemosensory gene expression in *Anopheles coluzzii* and *An. quadriannulatus* antennae

**DOI:** 10.1186/s13071-020-04085-3

**Published:** 2020-04-22

**Authors:** Giridhar Athrey, Zachary Popkin-Hall, Luciano Veiga Cosme, Willem Takken, Michel Andre Slotman

**Affiliations:** 1 Department of Poultry Science, Texas A&M University, College Station, TX USA; 2 Department of Entomology, Texas A&M University, College Station, TX USA; 3 Department of Ecology and Evolutionary Biology, Yale University, New Haven, CT USA; 4 Laboratory of Entomology, Wageningen University and Research, Wageningen, The Netherlands

**Keywords:** Anopheles, Chemosensation, Olfaction, Host seeking, Mating

## Abstract

**Background:**

Olfactory cues drive mosquito behaviors such as host-seeking, locating sugar sources and oviposition. These behaviors can vary between sexes and closely related species. For example, the malaria vector *Anopheles coluzzii* is highly anthropophilic, whereas *An. quadriannulatus* is not. These behavioral differences may be reflected in chemosensory gene expression.

**Methods:**

The expression of chemosensory genes in the antennae of both sexes of *An. coluzzii* and *An. quadriannulatus* was compared using RNA-seq. The sex-biased expression of several genes in *An. coluzzii* was also compared using qPCR.

**Results:**

The chemosensory expression is mostly similar in the male antennae of *An. coluzzii* and *An. quadriannulatus,* with only a few modest differences in expression. A handful of chemosensory genes are male-biased in both species; the highly expressed gustatory receptor *AgGr33*, odorant binding proteins *AgObp25*, *AgObp26* and possibly *AgObp10.* Although the chemosensory gene repertoire is mostly shared between the sexes, several highly female-biased *AgOrs*, *AgIrs*, and one *AgObp* were identified, including several whose expression is biased towards the anthropophilic *An. coluzzii*. Additionally, the expression of several chemosensory genes is biased towards *An. coluzzii* in both sexes.

**Conclusions:**

Chemosensory gene expression is broadly similar between species and sexes, but several sex- biased/specific genes were identified. These may modulate sex- and species-specific behaviors. Although the male behavior of these species remains poorly studied, the identification of sex- and species-specific chemosensory genes may provide fertile ground for future work.
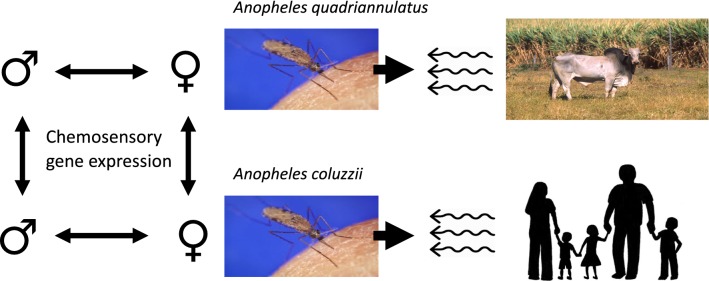

## Background

*Anopheles coluzzii* mosquitoes, one of the primary vectors of malaria in sub-Saharan Africa, depend on olfactory cues for host-seeking, identifying sources for sugar meals, as well as locating oviposition sites [1]. The behavior of female *An. coluzzii*, which are strongly attracted to the kairomones produced by humans [2], is in contrast to that of *An. quadriannulatus* females, a zoophilic sibling species that is attracted to cow odor [3]. The role of olfaction in the behavior of males of these species is less well understood. However, we do know that male *An. coluzzii* are attracted to plant volatiles [4], and that their chemoreceptor repertoire overlaps substantially with that of females [5]. Whether males of these species are attracted to host odors, if they share the preferential attraction to hosts observed in females, or if olfaction plays a role in mate recognition, is not known.

The antennae and the maxillary palps comprise the primary olfactory tissues of *An. coluzzii.* These appendages are covered densely with sensilla that house the olfactory sensory neurons which can express one of several classes of receptors: the olfactory receptors (*ORs*) or ionotropic receptors (*IRs*) (reviewed in [6]), and the gustatory receptor genes that encode the CO_2_ receptor (*AgGr22*, *AgGr23* and *AgGr24*) that are expressed in the maxillary palps in mosquitoes [7].

ORs are heteromeric ligand-gated ion channels encoded by the highly conserved co-receptor *AgOrco* and one of the remaining *AgOrs*. AgORs differ in their tuning breadth, ranging from narrowly to broadly tuned, but many of them respond to aromatics and heterocyclics [8–11]. While ORs function as ligand-gated ion channels, there are indications that they are modulated indirectly by G-protein signaling [12, 13].

IRs are also heteromeric ligand-gated ion channels, but these can contain up to three different subunits that include one or two of the broadly expressed co-receptors *AgIr25a*, *AgIr8a* and possibly *Or76b* [14, 15]. In addition to these receptors, odorant-binding proteins (OBPs) play a crucial role in odorant recognition by transporting hydrophobic odorants through the hemolymph (reviewed in [16]). OBPs are thought to be expressed in support cells and secreted in the hemolymph. A similar function is postulated for some chemosensory proteins (CSPs) [17]. Finally, a host of odorant degrading enzymes, such as several esterases and cytochrome P450’s are important for terminating signal transduction [16].

Differences in olfaction driven behavior between closely related species may be reflected in the structural and/or expression differences in the underlying chemosensory genes. In *Drosophila,* chemosensory gene expression has diverged considerably between closely related species with varying levels of host specialization [18]. In *Aedes aegypti*, divergence in both the expression and odor sensitivity of *AaegOr4* between the domestic and sylvatic subspecies has been linked to differential attraction to human odor [19]. Similarly, expression studies of the female antennae and maxillary palps of the anthropophilic *An. coluzzii* and zoophilic *An. quadriannulatus* have identified species-specific patterns of chemosensory gene expression, which may underlie diverging host preference [20, 21].

Chemosensory gene expression in olfactory tissues has been compared between females and males in *An. coluzzii* [5, 22, 23], as well as *Ae. aegypti* [24]. *Aedes aegypti* males depend on attraction to the human hosts to locate blood-meal seeking females to mate with [25]. Therefore, chemosensory genes crucial to locating hosts are expected to be expressed in both sexes in that species. Whether *An. coluzzii* males use human odor in locating mates or mating sites is not clear. However, the chemosensory gene repertoire is mostly shared between the sexes even though some differences in chemosensory gene expression were observed [5]. Furthermore, Foster and Takken [4] showed that while males are significantly less attracted to human odors compared to females, a non-trivial proportion of males (10%) responded to human odor in a dual choice olfactometer when the alternative was clean air. This provides some evidence that male *An. coluzzii* may respond to host odor, which, for example could play a role in mating swarm formation close to human habitation [26].

Species- and sex-specific and/or biased patterns of chemosensory gene expression in the olfactory organs of *An. coluzzii* and *An. quadriannulatus* may also reflect differences in preferred oviposition sites, differential attraction to plant odors, or in the possible detection of hitherto undescribed mating pheromones. For example, chemosensory genes that are specific to (i.e. expressed only in one sex) or biased in (i.e. expressed at higher level in one sex) females, and are expressed at different levels between species may be candidates for underlying oviposition and/or host-seeking behavior. Similarly, male-biased genes, especially those that vary between species, could provide rich opportunities for further work on mate recognition in the *An. gambiae* complex.

Male mosquitoes remain dramatically understudied compared to females. However, as sterile and transgenic mosquito techniques have emerged as a potential vector control tool, there has been renewed interest in the behavior of male anopheline mosquitoes [27]. Ongoing and future work will undoubtedly provide additional insight into these important aspects of male mosquito biology. Therefore, we compared antennal chemosensory gene expression between males of *An. coluzzii* and *An. quadriannulatus,* and included previously published data [21] to identify sex- and species-specific patterns of chemosensory gene expression between *An. coluzzii* and *An. quadriannulatus.*

## Methods

### Mosquito rearing

All mosquitos used in this study came from colonies that were kept and raised in the insectary at Texas A&M University, College Station, Texas, USA. These laboratory strains were of *An. coluzzii* M form (GASUA) originally collected in Suakoko, Liberia, and *An. quadriannulatus* (SANUQA) established from female mosquitoes collected in South Africa. Ambient conditions in the insectary were maintained at 25 °C, a relative humidity of 75–85% and a 12:12 h light:dark photocycle. Colonies were maintained by feeding females on defibrinated sheep blood using a membrane feeding system. Larvae were maintained at densities of about 150 per container in about 3.8 l (1 gallon) of water, and fed with Tetramin^TM^ (Tetra, Blacksburg, VA, USA) brand fish food. Pupae were collected each day and placed in gallon-sized adult rearing cages containing about 200–300 individuals. Males and females were kept together and fed a 5–10% sucrose solution for six to eight days until antennal dissections.

### Antennal dissection and RNA isolation

Six- to eight-day-old male mosquitoes were euthanized shortly after the start of the dark cycle by placing them at − 20 °C for 5 min. Once the mosquitoes were immobilized, they were placed on dry ice, and their antennae were dissected and stored in RNAlater (Life Technologies, Grand Island, NY, USA) at 4 °C for 24 h. The next day, the samples in RNAlater were stored at − 80 °C until RNA extraction. Antennae were dissected from 200–300 males per replicate. Two replicate samples were collected for each species.

Total RNA was isolated from each sample using miRNeasy columns according to the protocol supplied by Qiagen. RNA quantity was estimated using a Qubit fluorometer (Life Technologies), and a NanoDrop spectrophotometer. RNA quality was assessed using RNA Pico LabChip analysis on an Agilent BioAnalyzer 2100 by the AgriLife Genomics Center at Texas A&M University. For each replicate, approximately 1 μg of total RNA was used to prepare mRNA libraries for sequencing.

mRNA was isolated from total RNA and cDNA libraries were prepared using an Illumina TruSeq RNA Library kit. Each single-end sequencing library contained two replicates that were given a unique barcode sequence supplied by the library kit. The libraries were sequenced over two lanes of Illumina HiSeq 2000 in single end mode. Preparation and sequencing of libraries were both performed by Texas A&M AgriLife Genomics and Bioinformatics Services. Approximately 50–70 million reads with an average read length of 51 bp were generated for each replicate sample and used for further analysis.

### RNAseq analysis

Quality of the Illumina reads was assessed using FASTQC (ver 0.10.0). Sequencing reads were mapped to the reference *An. gambiae* PEST genome (AgamP4; https://www.vectorbase.org/organisms/anopheles-gambiae/pest, downloaded January 2020) using the software package STAR (version 2.7). No *An. quadriannulatus* reads were discarded for too many mismatches, despite mapping to the *An. gambiae* genome. Uniquely mapped reads in sorted BAM format were processed via the SpltNCigarReads tool from the Genome Analysis Toolkit [28], then used to estimate counts mapping to exon features using the featureCounts tool from the Subread package [29]. Tests for differential expression were performed in the R package *EdgeR* [30, 31]. Following normalization for each library, genes with CPM < 1, were excluded from the analyses. Next, we estimated common and tagwise dispersion, followed by statistical tests for significance using the ‘exactTest’ function. Genes were considered to be differentially expressed at FDR < 0.05, adjusted for multiple testing [32]. Transcripts per million (TPM) was calculated following the method described in Wagner et al. [33]. Negative Log_2_FC values were converted to negative fold change (FC) values using the equation FC = −(2^-Log^_2_^FC^).

To determine the functional roles of differentially expressed genes, we performed an analysis of molecular function and assignment of these genes to protein classes using the gene ontology database PantherDB (htpps://www.pantherdb.org). For each comparison, we separately analyzed the genes that were either significantly up-regulated in *An. coluzzii* or in *An. quadriannulatus*. The list of ENSEMBL gene ID’s for DE genes was exported to PantherDB and analyzed, using the *Anopheles gambiae* database as the reference. The output provides a list based on the percentage of DE genes that were assigned to a given molecular function, and similarly for the protein class analyses. The list was sorted in descending order of percentage hits to a given molecular function (or protein class). The top molecular functions and protein classes identified are reported.

### Quantitative PCR

Total RNA was extracted from pooled samples of male and female *An. coluzzii* using the Qiagen RNEasy® kit, incorporating on-column DNAse treatment using Qiagen® DNase I. RNA concentration and purity were calculating using a BioTek® Epoch™ microplate spectrophotometer and a BioTek® Take3 plate. Primers and dual-labeled probes were designed using Primer3Plus (Additional file 1: Table S1). All probes contained a FAM fluorophore and a TAMRA quencher. New primers and a probe were also designed in Primer3Plus for the established housekeeping gene *Rps7*. QPCR was performed using the SensiFAST™ Probe No-ROX One-Step Kit (Bioline) on a Bio-Rad® CFX96 thermocycler. Cycling was performed according to manufacturer recommendations, with reverse transcription at 45 °C for 10 min, polymerase activation at 95 °C for 2 min, followed by 40 cycles of denaturation at 95 °C for 5 s and annealing/extension at 60 °C for 20 s. Three replicate 20 μl reactions were performed with both male and female RNA for each gene. The average C_q_ value of each gene was calculated for both sexes. Relative gene expression levels and fold changes were calculated *via* the ΔΔC_q_ method [34].

## Results

The Illumina sequencing generated between 47.2 to 67.1 million reads per library for the male antennae samples. The total reads generated for the female antennae libraries ranged between 35.2 and 53.5 million [21]. For the male antennal samples, between 88.4 and 89.5% of the total reads mapped uniquely to exon features across the two species (Additional file 2: Table S2). Minimum quality score was 33 across the entire length of the reads for each library. After application of the filtering step to exclude genes with abundance lower than one TPM (transcript per million, applied to both male and female datasets), we retained a final set of 12,329 genes out of a total of 13,796 genes found in the annotation set. All further statistical comparisons were carried out on expression data from these genes. A second filtering step was applied in our discussion of the data in which we only consider receptors with TPM > 5, and *Obps* with TMP > 50.

### Chemosensory gene expression in *An. coluzzii vs An. quadriannulatus* male antennae

**Fig. 1:**
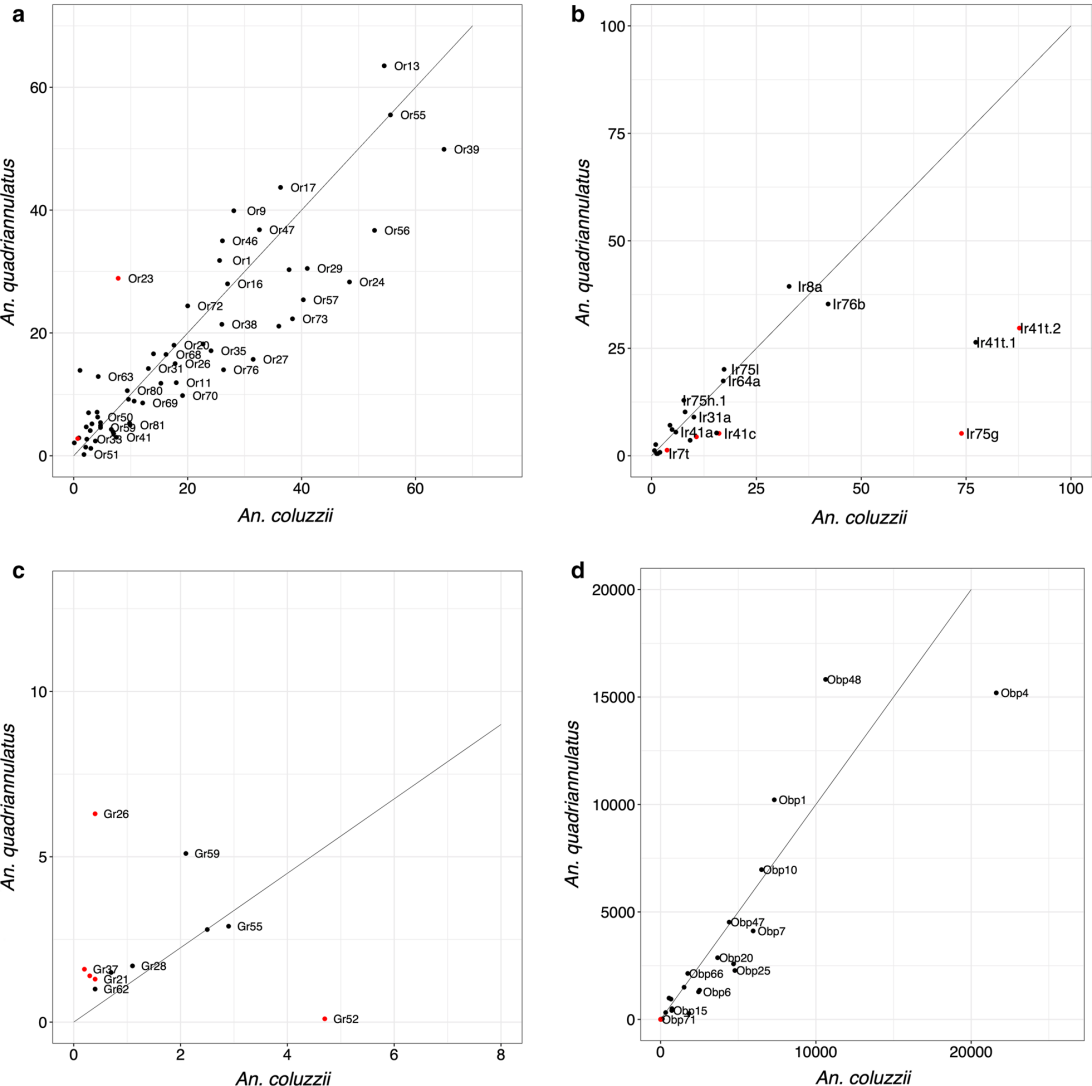
Chemosensory gene expression in male antennae of *An. coluzzii vs An. quadriannulatus.***a***Ors*. **b***Irs.***c***Grs.***d***Obps.* The line indicates equal expression between the two species. In **a**, *Orco* was excluded. In **b**, *Ir25a* was excluded. In **c**, *Gr33* was excluded. Red dots indicate significantly differentiated expression between samples

A relatively small number of genes (*n* = 536) were significantly differentially expressed between *An. coluzzii* and *An. quadriannulatus* male antennae (Additional file 3: Figure S1a, Additional file 4: Data S1). Of these, 286 were enhanced in *An. coluzzii, vs* 250 in *An. quadriannulatus.* Chemosensory gene expression was very similar between species, with only a handful of genes showing significantly enhanced expression. Overall, *Or* expression was highly correlated between the males of both species (*R*^2^ = 0.82, excluding *Orco*, Fig. 1a), with the total expression of the specific *Ors* comparable between species (1447.5 *vs* 1523.4 TPM). Of the *Ors* expressed > 5 TPM, only *Or23* shows DE (differential expression). The expression of this gene was 4.7-fold enhanced in *An. quadriannulatus* (Table 1), with relatively low expression in *An. coluzzii* (7.8 TPM). This corresponds with previous observations of very low expression of this gene in male *An. coluzzii* antennae [5].

**Table 1:** Chemosensory genes differentially expressed in the male antennae of *Anopheles coluzzii* vs. *An. quadriannulatus*

Gene ID	Gene	*An. coluzzii*	*An. quadriannulatus*	FC	log2FC	FDR
AGAP007797	Or23	7.8	28.9	− 3.83	− 1.94	0.0007
AGAP013085	IR75g	73.9	5.2	14.91	3.90	< 0.0001
AGAP012951	IR41c	16.1	5.2	2.92	1.55	0.0428
AGAP012969	IR41t.2	87.7	29.7	2.86	1.52	0.0287
AGAP007498	IR75k	10.7	4.4	2.39	1.26	0.0437
AGAP006717	Gr26	0.4	6.3	− 14.90	− 3.90	< 0.0001

*Notes*: Fold change and Log2 fold change are indicated as a positive value if expression is enhanced in *An. coluzzii* and *vice versa*. Only *Ors*, *IRs* and *Grs* expressed > 5 TPM, and *Obps* expressed >50 TPM are included

*Ir* expression between *An. coluzzii* and *An. quadriannulatus* males was highly correlated when the highly expressed co-receptor gene *Ir25* was included (*R*^2^ = 0.788), but in contrast to the *Ors*, was quite a bit lower if *Ir25* was excluded (*R*^2^ = 0.399, Fig. 1b). Total *Ir* expression was somewhat higher in *An. coluzzii* males (649.5 *vs* 466.2 TPM). The expression of *Ir75g*, *Ir41t.2*, *Ir41c* and *Ir75k* were significantly different, and between 2.4 and 14.9-fold enhanced in *An. coluzzii*. However, based on a comparison between *Ir* expression in male *An. coluzzii* antennae in this study with that of Pitts et al. [5], the abundances of *Ir75g* and *Ir41t.2* are relatively higher in our data set (Additional file 5: Figure S2).

The overall level of antennal *Gr* expression was similar in males of both species (343.2 *vs* 387.0 TPM) and was dominated by *Gr33,* which was expressed at a similarly high level in both species (327.4 *vs* 361.4 TPM, Fig. 1c). The correlation between *Gr* expression was therefore very high between the two species (*R*^2^ = 0.999), but disappeared entirely when *Gr33* is removed and only a few very lowly expressed *Grs* remained (*R*^2^ = 0.012). Of the *Grs* expressed at > 5 TPM only *Gr26* showed DE between the two species, with a 14.9-fold higher level expression in *An. quadriannulatus*. However, even in this species this gene was expressed at low levels (6.3 TPM), casting some doubt about the biological relevance of this observation.

Total *Obp* expression was similar in *An. coluzzii* male antennae (82,831 *vs* 74,7404 TPM), with a relatively high correlation (*R*^2^ = 0.84, Fig. 1d). No *Obp* expressed at TPM > 50 showed DE between species. *Obp26*, was detected at 7.3-fold higher levels in *An. coluzzii* males, but the difference was not significant (FDR = 0.116).

The most common molecular functions of the genes with enhanced expression in either species were ‘Binding’, ‘Catalytic activity’, and ‘Transporter activity’. The most common protein classes among the DE genes in both species were Hydrolase (PC00121), Oxidoreductase (PC00176), and Transferase (PC00220). Olfactory receptors or transmembrane signaling molecules were not among the significantly overrepresented groups in either species.

### Chemosensory gene expression in *An. coluzzii* male *vs* female antennae

**Fig. 2:**
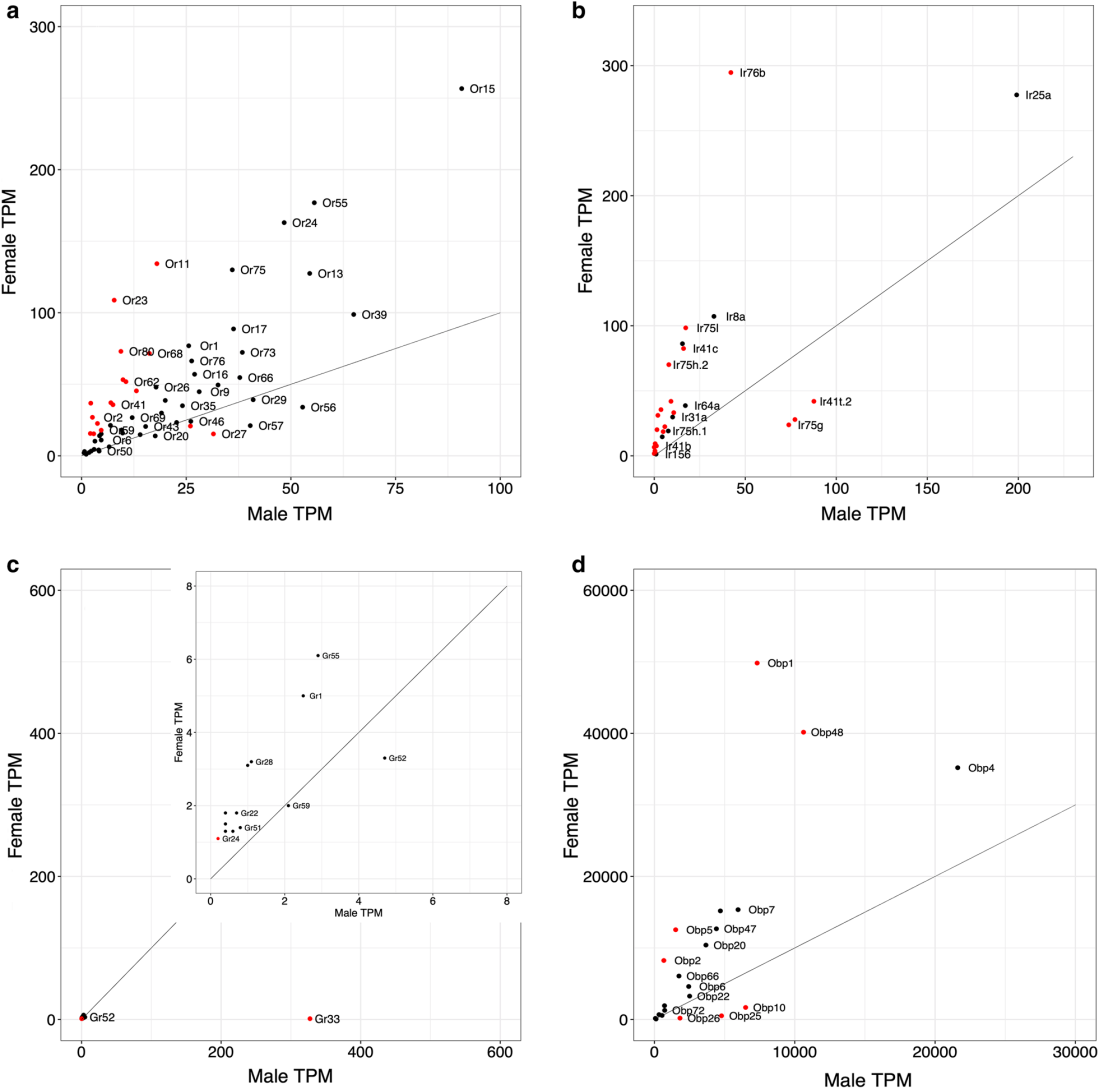
Chemosensory gene expression in male *vs* female antennae of the anthropophilic *An. coluzzii.***a***Ors*. **b***Irs.***c***Grs.***d***Obps.* The line indicates equal expression between the two species. In **a**, *Orco* was excluded. Red dots indicate significantly differentiated expression between samples

A total of 4664 genes with TPM > 1 showed DE between the antennae of females and males of this species (Fig. 2a). Of these, 2265 were enhanced in male antennae and 2399 were female-biased (Additional file 3: Figure S1b, Additional file 6: Data S2). A total of 59 specific *Ors* were expressed in the antennae of male *An. coluzzii* at TPM > 1, although 19 of these were expressed at low levels (TPM < 5). In the female antennae 63 specific *Ors* were expressed (TPM > 1). Overall, *Or* expression was much lower in the *An. coluzzi* males (1168.9 TPM) *vs* females (2752.1 TPM) and this was true for *Orco* expression as well (278.9 *vs* 1916 TPM). This is not surprising given the greater number of sensilla on the female antennae [35]. The correlation between *Or* expression in male and female antennae was intermediate (*R*^2^ = 0.566, excluding *Orco*, Fig. 2a). A total of 18 *Ors* showed DE between males and females, with all of them expressed > 5 TPM in at least one sex (Table 2). Not surprisingly, most of these were enhanced in females, with only two genes showing DE in males; *Or27* and *Or38* (3.3 and 2.0-fold respectively). However, these were expressed at similar levels in the sexes in a previous study [5].

**Table 2:** Chemosensory genes differentially expressed between the antennae of male and female *Anopheles coluzzii*

Gene ID	Gene	Male	Female	FC	log2FC	FDR
AGAP004355	Or27	31.5	15.3	3.27	1.71	0.0011
AGAP002640	Or38	26.0	20.8	2.01	1.01	0.0389
AGAP004974	Or31	13.1	45.4	− 2.19	− 1.13	0.0239
AGAP002995	Or59	4.7	17.9	− 2.46	− 1.30	0.0207
AGAP009704	Or68	16.2	71.6	− 2.77	− 1.47	0.0113
AGAP011978	Or62	10.6	51.8	− 3.03	− 1.60	0.0006
AGAP000226	Or41	7.5	35.7	− 3.22	− 1.69	0.0215
AGAP002044	Or77	2.9	15.4	− 3.34	− 1.74	0.0004
AGAP009520	Or10	7.0	37.1	− 3.46	− 1.79	0.0014
AGAP013512	Or81	9.9	53.2	− 3.69	− 1.89	0.0374
AGAP008114	Or22	3.8	22.6	− 3.71	− 1.89	0.0010
AGAP002560	Orco	278.9	1916.0	− 4.46	− 2.16	0.0007
AGAP011631	Or11	18.0	134.3	− 4.72	− 2.24	< 0.0001
AGAP001012	Or36	2.1	15.6	− 4.86	− 2.28	0.0001
AGAP005495	Or80	9.4	73.0	− 5.17	− 2.37	0.0016
AGAP009519	Or2	2.6	26.9	− 6.57	− 2.71	< 0.0001
AGAP007797	Or23	7.8	108.8	− 8.97	− 3.16	< 0.0001
AGAP003053	Or45	2.2	36.8	− 10.73	− 3.42	< 0.0001
AGAP013085	Ir75g	73.9	23.8	5.23	2.39	0.0014
AGAP004432	Ir41t.1	77.3	27.9	4.38	2.13	0.0013
AGAP012969	Ir41t.2	87.7	41.9	3.30	1.72	0.0019
AGAP007498	Ir75k	10.7	33.2	− 2.00	− 1.00	0.0369
AGAP008511	Ir21a	4.9	18.6	− 2.50	− 1.32	0.0428
AGAP002904	Ir41a	5.8	22.4	− 2.62	− 1.39	0.0136
AGAP012951	Ir41c	16.1	82.5	− 3.37	− 1.75	0.0030
AGAP005466	Ir75l	17.3	98.4	− 3.79	− 1.92	0.0007
AGAP008759	Ir41b	1.2	7.6	− 4.29	− 2.10	0.0005
AGAP011968	Ir76b	42.1	294.7	− 4.68	− 2.23	0.0040
AGAP001812	Ir75h.2	8.0	70.1	− 5.42	− 2.44	0.0014
AGAP013416	Ir7w	3.7	35.5	− 6.41	− 2.68	< 0.0001
AGAP002763	Ir7t	1.5	20.1	− 8.67	− 3.12	< 0.0001
AGAP000140	Ir100a	2.0	31.1	− 10.42	− 3.38	< 0.0001
AGAP013285	Ir7u	0.5	9.3	− 12.39	− 3.63	< 0.0001
AGAP013363	Ir7i	0.0	6.6	− 97.95	− 6.61	< 0.0001
AGAP000256	Ir93a	9.2	41.9	− 2.99	− 1.58	0.0056
AGAP010195	Gr33	327.4	1.1	472.80	8.89	< 0.0001
AGAP012321	Obp26	1821.8	186.7	17.74	4.15	0.0023
AGAP012320	Obp25	4781.0	514.4	15.24	3.93	< 0.0001
AGAP001189	Obp10	6499.0	1679.7	6.11	2.61	0.0001
AGAP007286	Obp48	10624.6	40160.1	− 0.41	− 1.28	0.0257
AGAP029062	Obp1	7318.8	49826.4	− 0.23	− 2.13	0.0120
AGAP009629	Obp5	1513.8	12545.3	− 0.19	− 2.40	0.0018
AGAP003306	Obp2	667.7	8248.0	− 0.13	− 2.99	0.0004

*Notes:* Fold change and Log2 fold change are indicated as a positive value if expression is enhanced in males and *vice versa*. Only *Ors*, *Irs* and *Grs* expressed > 5 TPM, and *Obps* expressed > 50 TPM are included

Chemosensory genes whose expression is strongly biased or specific to females are of interest, as these may play a crucial role in finding hosts or oviposition sites. The *Ors* with the highest fold change difference between females and males were *Or2*, *Or23* and *Or45.* The expression of these genes was between 6.6 and 10.7-fold that observed in males and exceeds the approximately 4-fold difference that might be expected from the larger number of sensilla on the female antennae (Table 2). All three of these genes were previously also found to be highly enhanced in female antennae of this species [5].

The overall expression of *Irs* was also lower in males than in females (650.3 *vs* 1447.3 TPM), with the number of *Irs* expressed at TPM > 1 at 22 and 28 in males and females, respectively. *Ir* expression was somewhat less correlated between males and females than *Or* expression (*R*^2^ = 0.403, Fig. 2b). Three of these were significantly enhanced in *An. coluzzii* males: *Ir75g*, *Ir41t.1* and *Ir41t.2* (Table 2). They were also the most highly expressed *Irs* in this sex (73.9 < TPM > 87.7), with the exception of the co-receptor *Ir25a*, and were between 3.3 and 5.2-fold higher expressed in males. However, like the male enhanced *Ors*, these male enhanced *Irs* were not found to be male-biased previously [5].

Among the female-biased *Irs*, two are co-receptors (*Ir8a* and *Ir76b*). Of the remaining, *Ir7i* and *Ir7u* were exclusive to females although expressed at relatively low levels (< 9.3 TPM). Strongly female-biased *Irs* were *Ir7t*, *Ir7w* and *Ir100a*, all of which are more than 6.4-fold enhanced in females, and expressed at very low levels in males (< 3.7 TPM, Table 1). A previous study found these *Irs* to be highly female-biased and expressed at low levels in males as well [5]. A suite of additional *Irs* was recently annotated in the *An. gambiae* genome [36], but none of these are expressed in the antennae of either sex in this species.

Fifteen *Grs* were expressed at TPM > 1 in the antennae of either sex (6 in males *vs* 15 in females). However, only two of these are expressed above TPM > 5: *Gr33* in males and *Gr55* in females. The correlation between *Gr* expression between sexes was very low (*R*^2^ = 0.058), and overall *Gr* expression is much higher in male *An. coluzzii* antennae (345.2 TPM) *vs* females (34.1 TPM). This is entirely due to *Gr33*, which was expressed at very high level in males (32.4 TPM), whereas it was barely expressed in females (1.1 TPM), a highly significant difference (FDR < 0.0001, Table 2, Fig. 2c). This was also found in a previous study [5]. *Gr33* was the most highly expressed chemosensory receptor in the male antennae, exceeding even *Orco.* No other *Gr* was significantly enhanced in either sex. The newly added *Gr62* [36], was not expressed in either sex.

A total of 28 Obp*s* were expressed at TPM > 1 in male *An. coluzzii* antennae, *vs* 31 in females (33 total). The correlation of *Obp* expression between the two sexes was similar to that of the *Ors* with *R*^2^ = 0.59 (Fig. 2d). Like the *Ors* and *Irs*, overall *Obp* expression was considerably lower in male antennae (82,832 *vs* 220,630 TPM). Five *Obps* were significantly enhanced in males, but only three of these (*Obp10*, *Obp25* and *Obp26*) were highly expressed (> 1821.8 TPM). The expression of all three was highly male-biased (between 6.1–17.7-fold). While *Obp25* was found to be male-biased in the previous work by Pitts et al. [5] as well, this was not true for *Obp10* and *Opb26,* both of which were female-biased in that study.

Six *Obps* were significantly enhanced in female antennae, of which *Obp1*, *Obp2*, *Obp5* and *Obp48* were medium to highly expressed (> 8248 TPM) and more than 2.4-fold enhanced in females. Amongs these, *Obp2* stands out for being expressed 8.0-fold in females *vs* males, suggesting an important role in female-specific processes. All three were highly female-biased in the Pitts et al. [5] study as well.

We compared the expression of a small number of chemosensory genes between *An. coluzzii* male and female antennae using quantitative PCR (Table 3). First, we compared *Orco,* which was 3.9-fold higher expressed in females according to the qPCR results (Table 4). This is similar to our RNAseq results. The highly male-biased *Gr33* was highly male-biased (65.2-fold) according to our qPCR results as well. However, *Ir75g* was not confirmed as male-biased, with qPCR indicating that this gene is expressed at approximately equal levels in both sexes. We also examined *Obp26*, which the qPCR results also showed to be male-biased (2.6-fold), although less so than the RNAseq data. Finally, the lowly expressed *Ir7i* was examined as well. The qPCR data confirmed the female-biased expression of this gene, although the estimated fold-change was lower (1.6-fold).

**Table 3:** Chemosensory genes differentially expressed between the antennae of male and female *Anopheles quadriannulatus*

Gene ID	Gene	Male	Female	FC	log2FC	FDR
AGAP004355	Or27	15.7	6.9	2.34	1.23	0.0220
AGAP004354	Or26	15.0	36.6	− 2.43	− 1.28	0.0155
AGAP009640	Or1	31.8	93.7	− 2.95	− 1.56	0.0127
AGAP002044	Or77	4.1	11.9	− 3.02	− 1.59	0.0012
AGAP009519	Or2	7.0	21.6	− 3.11	− 1.64	0.0073
AGAP002560	Orco	426.5	1367.1	− 3.29	− 1.72	0.0067
AGAP011978	Or62	8.9	29.0	− 3.30	− 1.72	0.0002
AGAP011979	Or60	3.9	11.6	− 3.34	− 1.74	0.0006
AGAP008114	Or22	2.4	7.2	− 3.44	− 1.78	0.0022
AGAP007797	Or23	28.9	108.3	− 3.66	− 1.87	0.0003
AGAP005760	Or33	2.7	10.1	− 3.72	− 1.90	0.0002
AGAP001012	Or36	1.4	5.4	− 3.82	− 1.93	0.0011
AGAP011989	Or63	12.9	46.4	− 3.93	− 1.97	0.0028
AGAP009704	Or68	16.5	68.3	− 4.02	− 2.01	0.0006
AGAP013512	Or81	5.0	22.0	− 4.45	− 2.15	0.0210
AGAP011991	Or61	5.2	17.2	− 4.68	− 2.23	0.0014
AGAP009520	Or10	3.6	18.0	− 4.76	− 2.25	0.0001
AGAP011631	Or11	11.9	64.7	− 5.09	− 2.35	< 0.0001
AGAP011990	Or64	2.8	10.2	− 5.17	− 2.37	0.0011
AGAP005495	Or80	10.6	66.2	− 6.23	− 2.64	0.0005
AGAP000226	Or41	3.0	20.6	− 6.91	− 2.79	0.0002
AGAP012951	IR41c	5.2	14.6	− 2.93	− 1.55	0.0096
AGAP004969	IR75d	7.1	21.7	− 3.03	− 1.60	0.0086
AGAP009014	IR31a	9.0	28.8	− 3.06	− 1.61	0.0485
AGAP002904	IR41a	5.5	17.3	− 3.17	− 1.67	0.0035
AGAP011968	IR76b	35.3	126.3	− 3.47	− 1.79	0.0212
AGAP005466	IR75l	20.1	75.1	− 3.74	− 1.90	0.0008
AGAP013416	IR7w	1.3	6.4	− 4.41	− 2.14	0.0001
AGAP000256	IR93a	3.6	16.2	− 4.61	− 2.20	0.0001
AGAP000140	IR100a	0.8	5.5	− 6.35	− 2.67	0.0005
AGAP002763	IR7t	0.5	5.1	− 9.72	− 3.28	< 0.0001
AGAP010195	Gr33	361.4	0.3	584.31	9.19	< 0.0001
AGAP004114	Gr1	2.8	9.9	− 3.53	− 1.82	0.0403
AGAP012320	OBP25	2276.7	179.3	11.09	3.47	< 0.0001
AGAP001189	Obp10	6969.0	693.9	8.79	3.14	< 0.0001
AGAP012321	OBP26	283.6	32.2	7.28	2.86	0.0302
AGAP012331	OBP29	993.3	192.7	4.82	2.27	0.0003
AGAP007287	OBP47	4527.0	10238.7	− 2.53	− 1.34	0.0474
AGAP009629	OBP5	1498.1	6116.4	− 4.14	− 2.05	0.0076
AGAP003306	OBP2	948.2	4053.9	− 4.56	− 2.19	0.0088

*Notes*: Fold change and Log2 fold change are indicated as a positive value if expression is enhanced in males and *vice versa*. Only *Ors*, *IRs* and *Grs* expressed > 5 TPM, and *Obps* expressed > 50 TPM are included

**Table 4:** Comparison of chemosensory gene expression in *An. coluzzii* male vs. female antennae using qPCR

Gene ID	Gene	FC qPCR	FC RNAseq
AGAP002560	*Orco*	− 3.9	− 4.5
AGAP010195	*Gr33*	65.2	472.8
AGAP013363	*Ir7i*	− 1.6	− 98.0
AGAP013085	*Ir75g*	1.1	− 5.2
AGAP012321	*Obp26*	2.6	17.8

*Note*: Positive values indicate enhanced expression in males

We analyzed the gene ontology, molecular function and protein class membership of differentially expressed (DE) genes. In *An. coluzzi* females, the top molecular functions of the upregulated genes were the terms ‘Binding’, ‘Catalytic activity’ and ‘Transporter activity’. The same molecular functions were enriched among the genes upregulated in male antennae as well. The class of proteins involved in nucleic acid binding (PC00171) hydrolase activity (PC00121) had the highest membership based on upregulated genes. These patterns were similar between male and female antennae. Olfactory receptor activity was significantly overrepresent in male upregulated genes (Fisher’s exact test: FDR = 0.034), but somewhat surprisingly, not in female upregulated genes.

### Chemosensory gene expression in *An. quadriannulatus* male *vs* female antennae

**Fig. 3:**
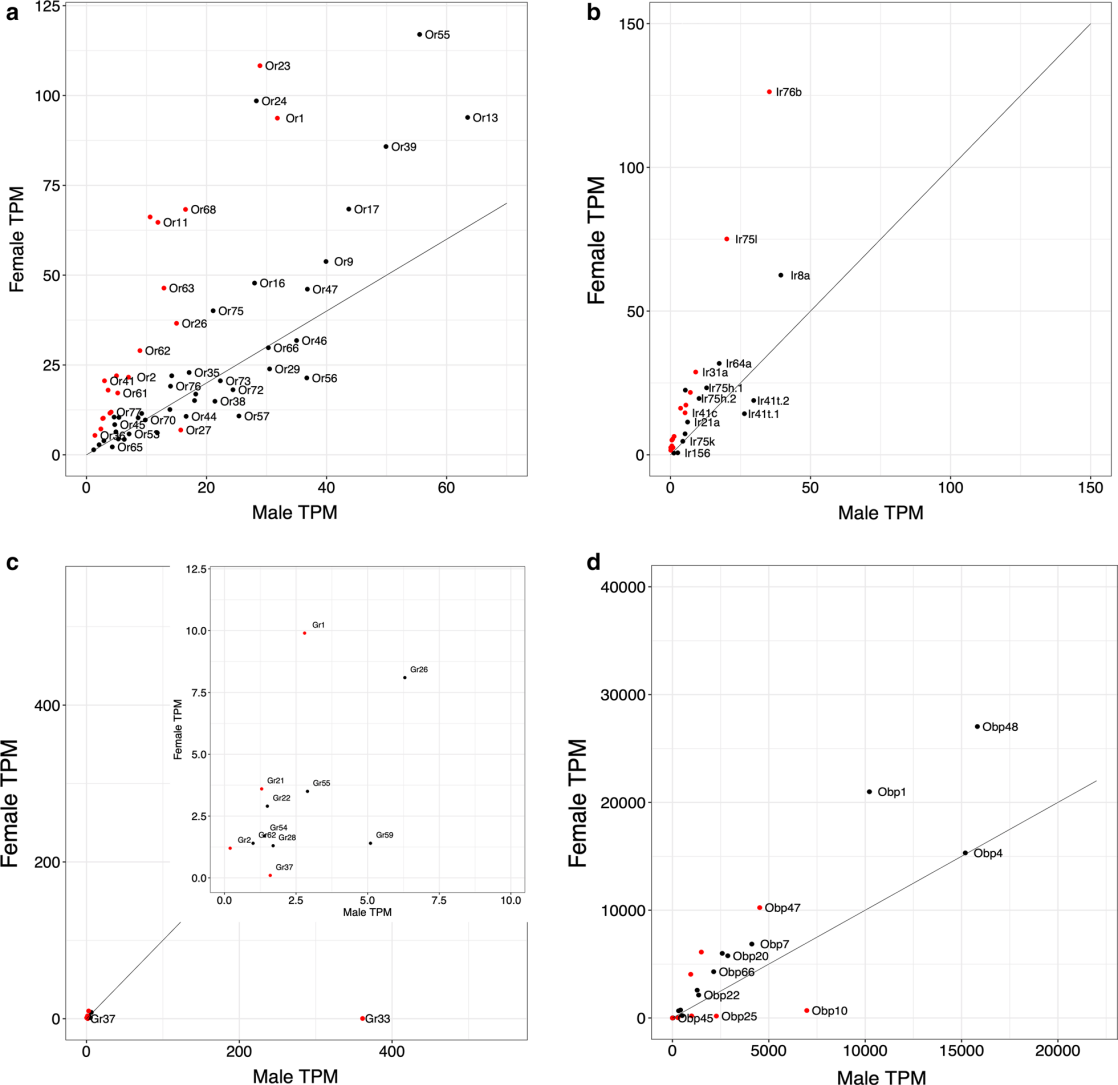
Chemosensory gene expression in male *vs* female antennae of the zoophilic *An. quadriannulatus.***a***Ors*. **b***Irs.***c***Grs.***d***Obps.* The line indicates equal expression between the two species. In **a**, *Orco* was excluded. In **b**, *Ir25a* was excluded. Red dots indicate significantly differentiated expression between samples

A total of 4043 genes expressed at TPM > 1 showed DE between female and male antennae. Of these, 1784 were enhanced in male antennae and 2259 were female-biased (Additional file 3: Figure S1c, Additional file 7: Data S3). Of the specific *Ors*, 60 were expressed at TMP > 1 in either sex and all were detected in both males and females. As in *An. coluzzii*, overall specific *Or* expression was considerably lower in antennae of males than in females (1096.7 TPM in males *vs* 1903.2 TPM in females), as is *Orco* expression (426.5 *vs* 1367.1 TPM, respectively). The correlation between overall specific *Or* expression was similar to that between *An. coluzzii* males and females (*R*^2^ = 0.68 excluding *Orco*, Fig. 3a). The expression of 20 specific *Ors* was significantly enhanced between sexes (Table 3, Fig. 3a). Of these, only the expression of *Or27* was enhanced significantly and 2.3-fold higher in males. This gene was also significantly enhanced in *An. coluzzii* males. Of the nineteen specific *Ors* whose expression was enhanced in females, the most highly enhanced *Ors* were *Or80* and *Or41* (6.2 and 6.9-fold respectively).

Twenty-one *Irs* were detected in male antennae at TPM > 1, *vs* 28 in females. As in *An. coluzzii*, overall *Ir* expression was lower in males (467.5 *vs* 792.1 TPM), although less so than in *An. coluzzii*. The correlation between male and female expression was relatively high (*R*^2^ = 0.84). No *Irs* were exclusively expressed in males (Table 3, Fig. 3b). Ten *Irs* were significantly enhanced in *An. quadriannulatus* females, but only *Ir7t* and *Ir100a* were enhanced more than 6-fold, beyond what might be expected to result simply from the larger number of sensilla on the female antennae [35]. However, both these genes were expressed at < 5.5 TPM, quite a bit lower than in the antennae of female *An. coluzzii* [21].

Only 11 *Grs* were expressed in the male antennae *vs* 10 in females at TPM > 1, with four expressed at TPM > 5 (Fig. 3c). Overall *Gr* expression in *An. quadriannulatus*, as in its sibling species, was much higher in male antennae than in females (387.1 *vs* 35.2 TPM), and the correlation between sexes was very low (*R*^2^ = 0.007). Like in *An. coluzzii* male antennae, *Gr33* was very highly expressed (361.4 TPM) and was the only *Gr* with significantly enhanced expression in males. As in *An. coluzzii*, *Gr33* was absent in females (< 1 TPM). No *Grs* were significantly enhanced in female antennae.

Thirty *Obps* were detected at TPM > 1 in male antennae *vs* 25 in females. As in *An. coluzzii*, overall *Obp* expression was much lower than in males than in females (74,403 *vs* 114,141 TPM, respectively), and the correlation between sexes was relatively high (*R*^2^ = 0.81) (Table 3, Fig. 3d). Four *Obps* expressed > 50 TPM were significantly enhanced in males (*Obp10*, *Obp25*, *Obp26* and *Obp29*), with their expression enhanced between 4.8–11.1-fold in both. The expression of three *Obps* (*Obp2*, *Obp5* and *Obp47*) is significantly between 2.5–4.6-fold enhanced in female antennae.

The analysis of molecular function and protein membership of these differentially expressed genes between the male and female *An. quandriannulatus* was broadly similar to patterns seen in *An. coluzzii.* In *An. quadriannulatus* females, the top molecular functions were the terms ‘Catalytic activity’ and ‘Binding’, and ‘Transporter activity’ followed by ‘Molecular transducer activity’. In the male antennae, catalytic activity and binding were important, and with structural activity and transporter activity third and fourth, respectively. Furthermore, nucleic acid binding (PC00171), hydrolase activity (PC00121), and transferase activity (PC00220) were the top three classes by membership in female antennae, whereas the top three were hydrolase, nucleic acid binding, and oxidoreductase (PC00176) in males. Transmembrane signaling receptor activity was overrepresented (Fisher’s exact test: FDR = 0.018) in the male-enhanced genes, but was absent from the molecular functions overrepresented in female-biased genes.

## Discussion

By comparing chemosensory gene expression between the sexes of closely related species with different host and/or oviposition site preferences we may be able to identify candidate chemosensory genes that modulate important mosquito behaviors. Oviposition and host-seeking behaviors are exclusive to females mosquitoes, whereas the attraction to flowers is shared between the sexes. Chemosensory genes whose expression is strongly enhanced in or exclusive to male antennae may modulate male-specific olfactory-driven behavior, although at present there is no evidence that olfaction plays a role in mate recognition within the *An. gambiae* complex [37].

It had previously been reported that the olfaction gene repertoire in the antennae is similar between the sexes of *An. coluzzii* [5]. We found this as well, with relatively high correlation between the expression of *Ors*, *Irs* and *Obps* in the sexes of both species. The high expression of *Gr33* in males of both species stands out. Nonetheless, some highly male- and female-biased genes were observed, some of which were also biased towards *An. coluzzii* or *An. quadriannulatus*.

While the male and female samples were processed by the same personnel using the same protocols, and were sequenced on the same platform around the same time, they were processed as separate batches, so batch effects may be present in the male-female comparisons. Therefore, we compared our results with previous data for *An. coluzzii* [5] to identify chemosensory genes that show consistent sex-specific expression. We also used qPCR on a small number of genes to confirm some of our results. Generally speaking, the chemosensory genes that were highly female-biased in our study were highly female-biased in the previous work by Pitts et al. as well [5], and the female-biased expression of two genes *Orco* and *Ir7i* was confirmed through qPCR.

In the male-biased genes however, there were some discrepencies. Five male-biased chemosensory genes were detected in both species in our study: *Or27*, *Gr33*, *Obp10*, *Obp25* and *Obp26*. While *Obp25* and *Gr33* were shown to be female-biased previously [5], this was not the case for the others. *Or27* was expressed around the same level in both sexes in that study and *Obp10* and *Obp26* were slightly or higly female-biased. However, of these genes the female-biased expression of *Gr33* and *Obp26* was examined and confirmed by qPCR. Therefore, at least some of the differences in expression observed between Pitts et al. [5] and our work may not be due to possible batch effects in our data.

Not much is known about the function of these genes, although *AgOr27* appears to be a narrowly tuned to the terpenes fenchone and carvone [11], both of which are abundant in several plant species. *Gr33* is the most highly expressed antennal chemosensory receptor gene, with the exception of *Orco*, in males of both species, but is all but absent from female antennae. Interestingly, the *Ae. aegypti* ortholog *AaGr19* is expressed at very low levels in the antennae and palps of both sexes [24, 38], so its function in male antennae may be specific to *Anopheles.* The homolog of this receptor in *Drosophila melanogaster* is *DmGr28*, which has several splice forms with non-olfactory function. Interestingly, *DmGr28b.c* is expressed in or near the Johnston’s organ, an auditory organ at the base of the antennae, and could play a role in sound perception. Another splice form, *DmGr28b(D),* modulates negative thermotaxis [39], and *DmGr28* also plays a role in larval dermal light detection [40]. Furthermore, it was recently shown that *DmGr28* modulates the attraction of *Drosophila* larvae to ribonucleosides [41].

None of the traits modulated by the *Drosophila* orthologs of *AgGr33* appear likely to be male-specific in mosquitoes. Although the detection of wingbeat frequencies plays a vital role during mosquito mating, females of several mosquito species were found to have a similar capacity to detect sound as males [42]. Heat gradient detection is modulated by *AgTRPA1* in the antennae of female *An. coluzzii* [43], which is expressed at comparable levels in the antennae of both sexes. Therefore, the function of *AgGr33* remains unclear.

The male-enhanced *Obp25* and possibly male-biased *Obp10* and *Obp26* are expressed in the mosquito body at much lower levels [5], consistent with a role in the olfactory system. Their high expression levels in the male antennae is all the more remarkable given the lower overall expression of *Obps* in male antennae. *Obps* are among the most highly expressed genes in sensory tissues and are thought to encode proteins that facilitate solubilization of hydrophobic volatiles into the hemolymph, and transport them to olfactory neurons. This model is supported by the observation that OBPs can bind odorant molecules [16] and that the addition of OBPs to heterologous expression systems increases sensitivity to odorants [44]. Besides, it has been speculated based on the large number of *AgObps vs AgOrs* expressed in mosquito palps, that *Obps* may also function as odorant sinks that prevent some odorants from reaching olfactory or ionotropic receptors [5]. Whether *Obp25* and *Obp26* have a male-specific function in the olfactory system is unknown, but given that *Obp25* and *Obp26* are expressed at different levels between *An. coluzzii* and *An. quadriannulatus* female palps [21] and that all three are expressed in other olfactory organs as well [5, 21, 45], indicates their function extends beyond that.

Unfortunately, the biology of male mosquitoes remains poorly studied [27], and little is known about what differences may exist in sensory perception between the males of these species. Conceivably, male *An. coluzzii* could use human odor as part of the various cues used to determine swarming site locations, which tend to be located inside or near villages [26]. A single study provided some support that *An. coluzzii* males are attracted to human odor [4], but this result needs confirmation. Furthermore, the expression of *AgOr1* and *AgOr8* in male antennae and palps, respectively [5, 7], suggests that males may be capable of detecting hosts. These two genes have been linked to vertebrate odorants in females [7, 8], although it should be noted that the detection of octenol by *Or8* could fulfill other roles, as the expression of this receptor is preserved in non-blood feeding *Toxorhynchites* mosquitoes [46]. Furthermore, if *An. coluzzii* males use some host odor cues, for example to locate their swarming sites near human habitations, it is not clear that they therefore share the host preference of females. As was shown previously and in this study, the repertoire of chemosensory genes largely overlaps between the sexes in both *An. coluzzii* [5] and *An. quadriannulatus*.

Among the chemosensory genes that are the focus of this study, only six are differentially expressed in *An. coluzzii vs An*. *quadriannulatus* male antennae. The expression of *Or23* and *Gr26* is enhanced in *An. quadriannulatus* males. This mimics the expression female antennae where the expression of both is significantly enhanced in *An. quadriannulatus* [21]. Of the four male *An. coluzzii-*biased genes, the expression of *Ir75k* and *Ir41c* are also enhanced in female antennae of this species [21]. The *An. coluzzii*-biased expression of *Ir75g* and *Ir41t.2* should be interpreted with caution, as these genes are represented at much higher relative levels in our data than in the previous work [5], and our qPCR data does not support the higher expression of *Ir75g* in *An. coluzzii* male *vs* females.

*Irs* have been linked to a variety of specific behaviors in *Drosophila. DmIr64a* in conjunction with the co-receptor *DmIr8a*, modulates acid avoidance behavior [47], and *DmIr84a* contributes to male courtship behavior *via* the detection of phenylacetic acid and phenylacetaldehyde. These two compounds are widely found in fruit and other plant tissues [48]. In *An. coluzzii*, *Ir41c* modulates the detection of amines, whereas *Ir75k* respondes to carboxylic acids, a class of compounds which includes major components of human sweat [49]. *Irs* play a role in host recognition in *Ae. aegypti*, as ORCO knock out mutants can still locate a host, although they lose their human host preference [50]. More recently it was shown that *Ae. aegypti* lacking the co-receptor *Ir8a* do not respond to lactic acid and other acidic volatiles, and have reduced attraction to human odor [51]. Recent work in *An. coluzzii* is consistent with the co-receptor *Ir8* being necessary for the detection of acids, whereas co-receptors *Ir25a* and *Ir76b* are needed for amine sensing [49].

If these *Anopheles* species make use of olfactory cues as part of the mate recognition process, the chemosensory genes underlying the detection of these cues may have diverged between males of the two species. The presence of contact sex pheromones to facilitate attraction to and recognition of conspecific mates has been supported in several mosquito species (reviewed in [37]), but data are lacking for the *An. gambiae* complex. It has been proposed that sex pheromones play a role in mate recognition between *An. coluzzii*, *An. gambiae* (*s.s.*) and other species, either in the form of a contact sex pheromone, or a low volatile pheromone acting when the sexes are in very close proximity [37]. Contact pheromones involved in mating have been inferred in other mosquito species. In *Aedes albopictus*, which can distinguish between conspecific and heterospecific females by touch, the prothoracic and mesothoracic tarsi have been implicated as the site where the pheromone is perceived [52]. One study proposed the presence of a volatile sex pheromone in *Culiseta inornata*, a species in which males mate with females shortly after emerging from the pupal stage [53], but a later study was not able to repeat this result [54].

Another group of chemosensory genes of interest are those genes that are (mostly) exclusive to female antennae, as this may indicate a role in host-seeking or oviposition. Antennae of female *An. coluzzii* contain approximately 2.9-fold more trichoid sensilla, which express *AgOrs* [35], than those in males, and other olfactory sensilla on the antennae are approximately 4-fold more abundant in females as well [55]. Overall olfactory genes expression is therefore significantly higher in female antennae [5], and this is what we found as well. This complicates the identification of female-biased chemosensory genes, but chemosensory genes enhanced more than might be expected based on the higher number of sensilla in females may be of interest.

Therefore, we focus here on genes > 6-fold enhanced in female antennae. In *An. coluzzii* this set includes three *Ors* (*Or2*, *Or23* and *Or45*) and five *Irs* (*Ir7w*, *Ir7t*, *Ir100a*, *Ir7u* and *Ir7i*) and *Obp2*. However, *Ir7u* and *Ir7i* are relatively lowly expressed even in females (6.6 TPM and 9.3 TPM), casting some doubt on their biological relevance. *Ir100a* and *Ir7t* are more than 6-fold enhanced in *An. quadriannulatus* females *vs* males as well, but interestingly *Ir7w*, *Ir7t* and *Ir100a* are between 2.5–3.4-fold higher expressed in the female antennae of *An. coluzzii vs An. quadriannulatus* [21]. Possibly, this indicates the involvement of these genes in species-specific differences in female behaviors, such as the human host preference of *An. coluzzii.* The expression of *Or45* is also significantly enhanced in *An. quadriannulatus*, but only 1.5-fold times.

Differential oviposition site preference between species could also result in female-biased chemosensory gene expression. The breeding sites of *An. coluzzii* result from human activity, and can consist of rice fields, drainage ditches, and reservoirs in savannah areas and urban pools in forested areas [56]. This differs from *An. gambiae* (*s.s.*), which prefers more rain-dependent and ephemeral habitats [57–59]. Not much information is available on the oviposition sites used by *An. quadriannulatus.* Larvae of this species have been collected from temporary pools adjacent to a river, suggesting their larval habitat is similar to that of *An. gambiae* (*s.s.*) [60]. They are also known to share larval habitats with *An. arabiensis* [61], although the volatiles used to identify preferred breeding sites may differ between the females of the two species [62].

## Conclusions

Our comparison of species and sex-specific chemosensory gene expression in the antennae of the anthropophilic malaria vector *An. coluzzii* and the zoophilic *An. quadriannulatus* has identified a small number of genes that show expression patterns that may underlie sex- and/or species-specific behavior. At the moment, a dearth of information on crucial aspects of the behavior of these species prevents a fuller interpretation of the results. Future and ongoing work on the attraction of the *Anopheles* males to host and and or swarming site odors, will assist in elucidation the relevance of the expression patterns observed here.

## Supplementary information

**Additional file 1: Table S1.** Primer and Probe sequences for qPCR.

**Additional file 2: Table S2.** Mapping statistics.

**Additional file 3: Figure S1.** Level of gene expression and LogFC between (**a**) male antennae of *An. coluzzii* and *An. quadriannulatus*, (**b**) male and female *An. coluzzii* antennae, and (**c**) male and female *An. quadriannulatus* antennae. Genes with significantly enhanced expression are indicated by a red dot.

**Additional file 4: Data S1.** Gene expression data for the male antennae of *An. coluzzii vs An. quadriannulatus.*

**Additional file 5: Figure S2.** Levels of *Ir* expression in *An. coluzzii* male antennae observed in this study *vs* that of *Pitts* et al. [5].

**Additional file 6: Data S2.** Gene expression data for *An. coluzzii* male *vs* female antennae.

**Additional file 7: Data S3.** Gene expression data for *An. quadriannulatus* male *vs* female antennae.

## Data Availability

The dataset(s) supporting the conclusions of this article are available in the NCBI Sequence Read Archive repository, BioProject ID PRJNA608544 (male data) and BioProject ID PRJNA400609 (female data). The gene expression data supporting the conclusions of this article are included with the article and its additional files.

## Abbreviations

CPM: Count per million
IR: Ionotropic receptor
GR: Gustatory receptor
OBP: Odorant binding protein
OR: Olfactory receptor
TMP: Transcripts per million
FC: Fold change
