# Supplementary material for: Species and sex-specific chemosensory gene expression in Anopheles coluzzii and An. quadriannulatus antennae
Source: Parasit Vectors. 2020 Apr 22;13:212. doi: 10.1186/s13071-020-04085-3 (PMC7178735; doi:10.1186/s13071-020-04085-3)
Supplement: Supplementary file 1 — Additional file 1: Table S1. Primer and Probe sequences for qPCR. [file 13071_2020_4085_MOESM1_ESM.pdf]

Table S1. Primer and probe sequences for qPCR.

Gene	Forward Primer	Probe	Reverse Primer
Gr33	GTGTCAACGGTAGCCGAAAT	TGCAGCTACGCCGAGGACTACTTCACG	GTGCGTCCAGCACGTAGTAA
Ir7i	TACCCGGCCTGTACTACACC	ATCCTGATGGGTGGACCGGGTCGG	CACCAGGCTGATGACGTAGA
Ir75g	AACGGTGTGATGCAGTACGA	CAAGCCAAGGTGTGCCGAAAATGAGGT	AACCTCCGGCATTTTAACCT
Obp26	ACAAGGGCGAAATTGATGAG	TCATCGAGAAGCTGTCGGTTGACCACG	TTCTTCACCAGTCCCTCCAC
Orco	ATAAGGCAACCAACGAGACG	GCAATGAGCGGACCGGCGTACATT	ACCATCGAAAACAGCAGGAA
Rps7	CGTGAGGTCGAGTTCAACAA	ACGTGCCGGTGCCGAAACAGAAGG	CACCAGACGGGTCTGTACCT
