# Supplementary material for: Species and sex-specific chemosensory gene expression in Anopheles coluzzii and An. quadriannulatus antennae
Source: Parasit Vectors. 2020 Apr 22;13:212. doi: 10.1186/s13071-020-04085-3 (PMC7178735; doi:10.1186/s13071-020-04085-3)
Supplement: Supplementary file 2 — Additional file 2: Table S2. Mapping statistics. [file 13071_2020_4085_MOESM2_ESM.pdf]

Table S2. Mapping statistics

Mapping stastistics	Total reads	Uniquely mapped	%	unmapped due to too many mismatch
CMA1	57,337,067	51,009,519	88.96%	0%
CMA2	47,219,860	42,259,374	89.49%	0%
QMA1	67,063,752	59,297,397	88.42%	0%
QMA2	63,501,733	56,785,144	89.42%	0%
CFA1	53,462,674	45,797,639	85.66%	0%
CFA2	35,277,933	32,478,353	92.06%	0%
QFA1	39,100,386	33,869,134	86.62%	0%
QFA2	38,079,310	15,179,946	39.86%	0%
average	50,130,339	42,084,563	82.56%	0%
