# Supplementary figures and images for: Species and sex-specific chemosensory gene expression in Anopheles coluzzii and An. quadriannulatus antennae
Source: Parasit Vectors. 2020 Apr 22;13:212. doi: 10.1186/s13071-020-04085-3 (PMC7178735; doi:10.1186/s13071-020-04085-3)

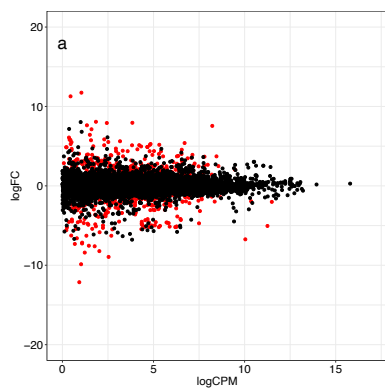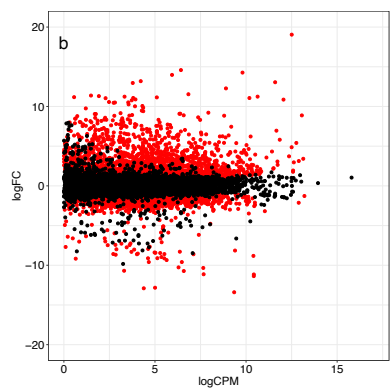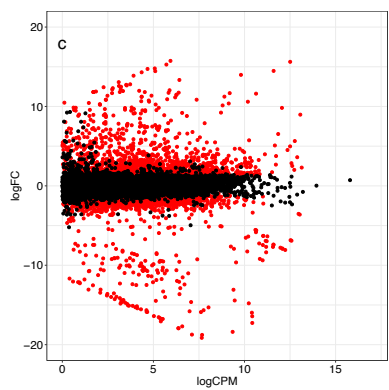

Supplement: Supplementary file 3 — Additional file 3: Figure S1. Level of gene expression and LogFC between (a) male antennae of An. coluzzii and An. quadriannulatus, (b) male and female An. coluzzii antennae, and (c) male and female An. quadriannulatus antennae. Genes with significantly enhanced expression are indicated by a red dot. [file 13071_2020_4085_MOESM3_ESM.pdf]

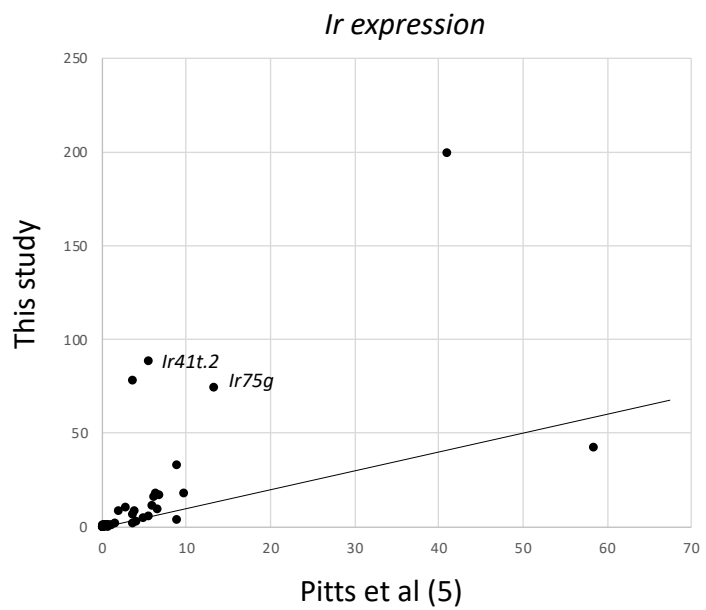

Supplement: Supplementary file 5 — Additional file 5: Figure S2. Levels of Ir expression in An. coluzzii male antennae observed in this study vs that of Pitts et al. [5]. [file 13071_2020_4085_MOESM5_ESM.pdf]
